# Silicon-Mediated Enhancement of Heavy Metal Tolerance in Rice at Different Growth Stages

**DOI:** 10.3390/ijerph15102193

**Published:** 2018-10-08

**Authors:** Fei Huang, Xiao-Hui Wen, Yi-Xia Cai, Kun-Zheng Cai

**Affiliations:** 1 Department of Ecology, College of Natural Resources and Environment, South China Agricultural University, Guangzhou 510642, China; 2 Key Laboratory of Tropical Agro-Environment, Ministry of Agriculture, South China Agricultural University, Guangzhou 510642, China

**Keywords:** phytotoxicity, heavy metal toxicity, physiological response, systemic uptake

## Abstract

Silicon (Si) plays important roles in alleviating heavy metal stress in rice plants. Here we investigated the physiological response of rice at different growth stages under the silicon-induced mitigation of cadmium (Cd) and zinc (Zn) toxicity. Si treatment increased the dry weight of shoots and roots and reduced the Cd and Zn concentrations in roots, stems, leaves and grains. Under the stress of exposure to Cd and Zn, photosynthetic parameters including the chlorophyll content and chlorophyll fluorescence decreased, while the membrane permeability and malondialdehyde (MDA) increased. Catalase (CAT) and peroxidase (POD) activities increased under heavy metals stress, but superoxide dismutase (SOD) activities decreased. The magnitude of these Cd- and Zn-induced changes was mitigated by Si-addition at different growth stages. The available Cd concentration increased in the soil but significantly decreased in the shoots, which suggested that Si treatment prevents Cd accumulation through internal mechanisms by limiting Cd^2+^ uptake by the roots. Overall, the phenomena of Si-mediated alleviation of Cd and excess Zn toxicity in two rice cultivars could be due to the limitation of metal uptake and transport, resulting in an improvement in cell membrane integrity, photosynthetic performance and anti-oxidative enzyme activities after Si treatment.

## 1. Introduction

Cadmium (Cd) can easily accumulate in rice plants through the apoplastic or symplastic pathways in the root system, leading to physiological damage such as stunted plant growth, reduced reproduction, degradation of chlorophyll and inactivation of enzymes [1]. Zinc (Zn) is an essential element for the health and development of rice, but the presence of excess Zn in soil is associated with highly toxic effects in plants, which leads to the inhibition of seed germination, root development and plant growth [2]. Therefore, it is important to develop reliable approaches to prevent the accumulation of these heavy metals in rice, which is a staple crop in several regions around the world, especially in Asia.

Silicon (Si) is a beneficial and possibly essential element for plants [3], which plays important roles in plant growth and development [4,5]. Moreover, there is mounting evidence that the application of Si to soils can alleviate Cd or Zn toxicity in many plant species, including rice [6,7,8], maize [9], wheat [10] and cotton [11]. Regarding rice plants, there is increasing evidence that Si application increases resistance to Cd toxicity via a variety of mechanisms. Nwugo and Huerta [12,13] demonstrated that the positive effect of Si on Cd resistance could be due to the inhibition of Cd uptake in roots and the enhancement of light-use-efficiency in leaves. Nwugo and Huerta, Lin et al. and Ma et al. [14,15,16] reported that the addition of Si to soil increased Cd tolerance in rice and could be attributed to the enhancement of anti-oxidative enzyme activities and the regulation of protein production. Furthermore, Liu et al. [17], He et al. [18] and Ma et al. [7] showed that the uptake of Cd in rice roots is inhibited by a hemicellulose-bound form of silicon in the cell walls. In comparison to the case with Cd, there are fewer reports of Si-mediated reduction of toxicity associated with excess Zn in rice plants. Gu et al. [5] showed that Si-mediated mitigation was due to a reduction of metal uptake and translocation, alongside binding of the metal in the cell wall. Song et al. [19] investigated the role of Si in enhancing tolerance to Zn in rice and attributed the improvement to greater membrane integrity, increased photosynthesis, and increased anti-oxidant defense capacity. These studies together with other investigations [20,21,22,23] highlighted the important roles of Si in Cd and Zn detoxification in rice plants. However, the physiological response of rice involved in these protective effects of Si during different growth phases remains poorly elucidated. Thus, there is much useful information yet to be gained from the examination of the reaction of rice plants exposure to Cd and excess Zn at different growth stages.

Our previous studies indicated that Si could ameliorate the toxicity of heavy metals in the two rice cultivars Feng-Hua-Zhan and Hua-Hang-Si-Miao, by its effects on root traits, cell structure and exudates [24]. Hence, the objectives of this study were: (1) to reveal Si-induced physiological alterations of two rice cultivars at different phenological stages under the Cd and excess Zn stress; and (2) to gain better insight into the possible mechanisms involved in the Si-mediated detoxification of Cd and excess Zn.

## 2. Materials and Methods

### 2.1. Plant Materials, Soil Sample and Experimental Treatments

Seeds of Feng-Hua-Zhan and Hua-Hang-Si-Miao rice cultivars were obtained from the Guangdong Academy of Agricultural Science and South China Agricultural University, respectively. Both cultivars are widely grown throughout China.

Uniform-sized rice seeds were surface sterilized with H_2_O_2_ (10%) for 10 min, then were rinsed and soaked in distilled water overnight, followed by germination in an incubator under moist conditions at 25 °C. When two to three leaves appeared, the healthy seedlings were transplanted into individual plastic pots (22 cm high × 22 cm diameter), which were then placed in a greenhouse where the temperature ranged from 25 to 35 °C and the relative humidity was 40–70%. The growth periods of the rice plant were divided into three stages: seedling, tillering and ripening. The plant samples at different growth stages were collected for subsequent chemical and biochemical analysis. 

The soil samples were collected from a paddy soil in the farm of South China Agricultural University, Guangzhou, China, in which the total Cd, Zn and Si concentrations were 0.78 mg·kg^−1^, 114.98 mg·kg^−1^ and 52.6 mg·kg^−1^, respectively, as determined by atomic absorption spectrometry (AAS), and the pH was 5.9. The soil was air-dried, crushed to be able to pass through a 2 mm sieve and then mixed in the nutrient solution prepared with 0.325 g·kg^−1^ N of urea, 0.243 g·kg^−1^ P of Ca(H_2_PO_4_)_2_, and 0.241 g·kg^−1^ K of KCl. 

Our previous study found that the optimal amount of Si addition for rice plant growth under Cd and excess Zn stress was 42 mg·kg^−1^. In the present work, seven treatments with four replicates each were designed for both rice cultivars: (1) CK (neither heavy metal nor silicon added); (2) Cd (Cd added at 50 mg·kg^−1^); (3) Zn (Zn added at 200 mg·kg^−1^); (4) Cd + Zn (50 mg·kg^−1^ Cd and 200 mg·kg^−1^ Zn); (5) Cd + Si (50 mg·kg^−1^ Cd and 42 mg·kg^−1^ Si); (6) Zn + Si (200 mg·kg^−1^ Zn and 42 mg·kg^−1^ Si); and (7) Cd + Zn + Si (50 mg·kg^−1^ Cd, 200 mg·kg^−1^ Zn and 42 mg·kg^−1^ Si). The Cd, Zn and Si were added as CdCl_2_·5H_2_O, ZnSO_4_·7H_2_O and K_2_SiO_3_·nH_2_O, respectively, in which additional K introduced by K_2_SiO_3_·nH_2_O was subtracted from KCl. The K_2_SiO_3_·nH_2_O, CdCl_2_·2.5H_2_O and ZnSO_4_·7H_2_O were dissolved in 100 mL of water and then mixed thoroughly with the soil in order to obtain a homogeneous distribution.

### 2.2. Chemical Analysis of Plant Tissues

Plant samples were separated into root, stem, leaf and/or grain, and then dried at 70 °C for 72 h. After drying, the plant tissues were ground and digested in a mixture of HNO_3_-HClO_4_ (5:1) for heavy metal analysis by AAS (Hitachi Z-200, Tokyo, Japan). The Si concentration was measured by the colorimetric molybdenum blue method described by Liang et al. [9].

### 2.3. Biochemical Analysis

#### 2.3.1. Electrolyte Leakage Measurement and MDA Content 

Fresh leaf samples were cut into small pieces and placed in test tubes containing 10 mL of distilled deionized water and emerged in a water bath at a constant temperature of 32 °C. The electrical conductivity was measured by an electrical conductivity meter (Hach 51910, Loveland, CO, USA) and the electrolyte leakage was calculated by the formula given by Kaya et al. [25]. The level of lipid peroxidation was quantitated as the amount of MDA determined by the thiobarbituric acid (TBA) reaction, following the method of Heath and Packer [26].

#### 2.3.2. Total Chlorophyll Content and Chlorophyll Fluorescence Parameters

Fully expanded leaves were collected and ground into a fine powder and then extracted with 10 mL of 80% acetone (v/v). The concentrations of chlorophyll a and b in the extract supernatants were determined by spectrophotometer after centrifugation, as described by Feng et al. [27]. The total chlorophyll content was calculated by the equation given by Porra et al. [28]. Chlorophyll fluorescence measurements were performed with a chlorophyll fluorometry (OS-3OP, Opti-Sciences, Boston, MA, USA). The maximum quantum efficiency of photosystem II (PSII) photochemistry (*F*_v_*/F*_m_) and the basal quantum yield (*F*_v_*/F*_0_) were determined by the methods previously described by Gao et al. [29].

#### 2.3.3. Anti-Oxidative Enzyme Activities 

Leaf fragments were ground in an ice-cold potassium phosphate buffer (pH 7.8) and then centrifuged at 12,000 r·min^−1^ for 20 min at 4 °C. The supernatant was stored at 4 °C for analysis of enzyme activity. Superoxide dismutase (SOD, EC 1.15.1.1), guaiacol peroxidase (POD, EC1.11.1.7) and catalase (CAT, EC 1.11.1.6) activities were assayed using the procedure described by Li et al. [30].

### 2.4. Statistical Analysis

All experimental data were expressed as the means plus or minus one standard deviation. Differences between the treatments were statistically examined by a one-way analysis of variance (ANOVA) and Duncan’s multiple range test (MRT) at a 0.05 probability level using SPSS 18.0 for Windows (SPSS Inc., Chicago, IL, USA).

## 3. Results

### 3.1. Effect of Si on Plant Growth 

**Figure 1:**
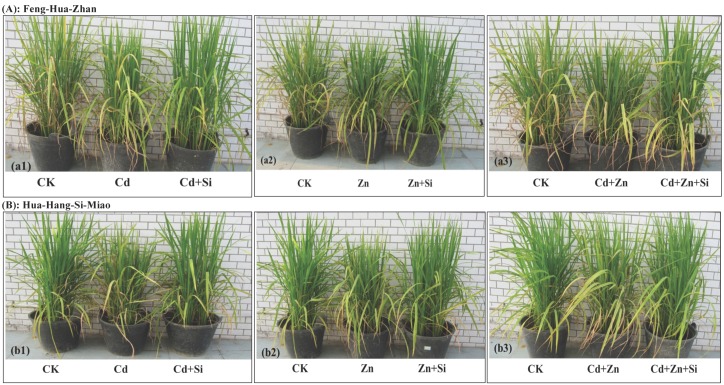
Mitigation of Si on growth inhibition in two rice cultivars under Cd and excess Zn stress at the tillering stage: (**a1**) and (**b1**) Cd stress; (**a2**) and (**b2**) Zn stress; (**a3**) and (**b3**) Cd + Zn stress.

Heavy metal treatments (Cd, Zn, Cd + Zn) significantly reduced the dry weight of the shoots and roots compared with the control (CK), while Si treatments (Cd + Si, Zn + Si, Cd + Zn + Si) dramatically increased the shoot and root weight compared to the non-Si treatments (Cd, Zn, Cd + Zn). As qualitatively depicted in Figure 1 and quantitatively displayed in Table 1, in the ripening stage, the shoot dry weight was 28% (Cd + Si vs. Cd), 27% (Zn + Si vs. Zn) and 61% (Cd + Zn + Si vs. Cd + Zn) higher in Feng-Hua-Zhan, and 48% (Cd + Si vs. Cd), 90% (Zn + Si vs. Zn) and 41% (Cd + Zn + Si vs. Cd + Zn) higher in Hua-Hang-Si-Miao. In the tillering stage, the comparable increases in Feng-Hua-Zhan were 31%, 57%, and 16% for the root dry weights, and 28%, 27%, and 11% for the shoot dry weight. 

These results show the beneficial effect of Si on plant growth under Cd and excess Zn stress.

### 3.2. Effect of Si on Cd and Zn Accumulation in Rice Plants

**Figure 2:**
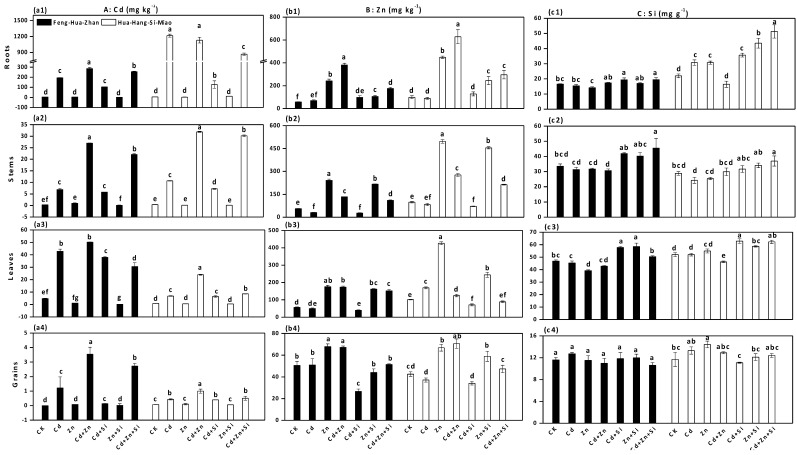
Effect of Si on element contents in different rice organs at the ripening stage: (**a1**), (**b1**) and (**c1**) roots; (**a2**), (**b2**) and (**c2**) stems; (**a3**), (**b3**) and (**c3**) leaves; (**a4**), (**b4**) and (**c4**) grains. Error bars indicate standard deviations (*n* = 4). Bars with different letters are significantly different (*p* < 0.05) according to Duncan’s MRT.

**Figure 3:**
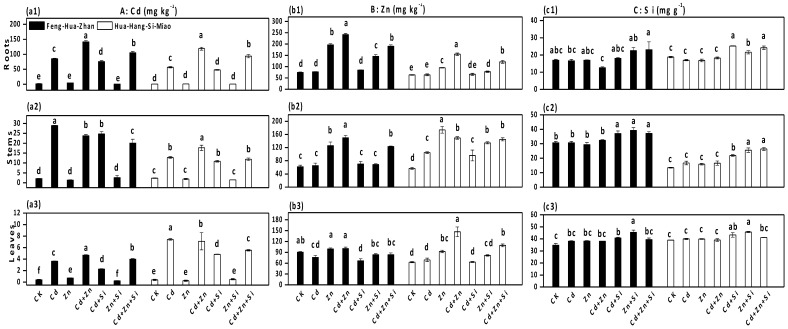
Effect of Si on elements content in different rice organs at the tillering stage: (**a1**), (**b1**) and (**c1**) roots; (**a2**), (**b2**) and (**c2**) stems; (**a3**), (**b3**) and (**c3**) leaves. Error bars indicate standard deviations (*n* = 4). Bars with different letters are significantly different (*p* < 0.05) according to Duncan’s MRT.

Si addition was efficient in reducing Cd and Zn uptake by rice in the ripening stage (Figure 2). In Feng-Hua-Zhan rice, the Cd concentrations in plant tissues (root, stem, leaf and grain) decreased by 47%, 16%, 11% and 89% (Cd + Si vs. Cd), and 11%, 18%, 39% and 23% (Cd + Zn + Si vs. Cd + Zn), respectively (Figure 2a). There was a sharp decline in the average Cd concentration in grains (from 1.2 ± 0.8 to 0.1 ± 0.01 mg·kg^−1^) when high Cd-stressed plants were grown in Si-supplied soil. In addition, the Zn concentrations in rice tissues (root, stem, leaf and grain) increased significantly under Zn alone treatment, whereas Zn content with Si treatments decreased in Feng-Hua-Zhan rice by 56%, 11%, 35% and 8% (Zn + Si vs. Zn) and decreased in Hua-Hang-Si-Miao rice by 45%, 8%, 43% and 12% (Cd + Zn + Si vs. Cd + Zn) (Figure 2b). In short, the protective effect of Si on Cd and Zn accumulation was also observed in the tillering stage (Figure 3).

Accumulations of Cd and Zn in different rice organs were not identical, but they consistently followed the order: root > stem or leaf > grain under all treatments. The distribution for Zn in the Zn-stressed plants grown in Si-treated soil was: stem > root or leaf > grain. In comparison, the distribution characteristics of Si were generally in the following order: leaf > root or stem > grain (Figure 2c).

### 3.3. Effect of Si on Plasma Membrane Permeability and MDA Content 

**Figure 4:**
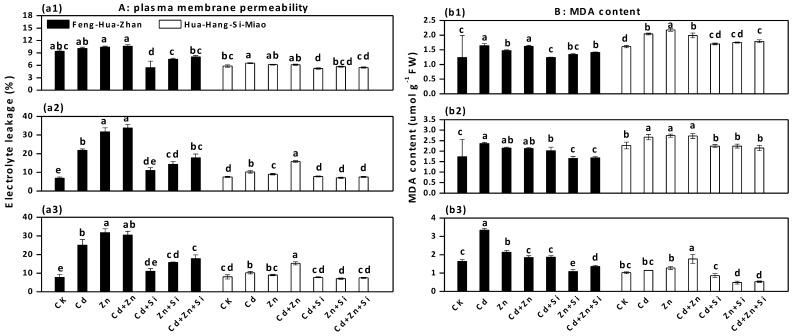
Effect of Si on plasma membrane permeability and MDA content in two rice cultivars at different growth stages: (**a1**) and (**b1**) seedling stage; (**a2**) and (**b2**) tillering stage; (**a3**) and (**b3**) ripening stage. Error bars indicate standard deviations (*n* = 4). Bars with different letters are significantly different (*p* < 0.05) according to Duncan’s MRT.

The toxic effects of Cd and excess Zn on plasma membrane permeability were evident in comparison to that of the control (CK), but these effects were mitigated by Si addition in both rice cultivars during all three growth phases (Figure 4a). In the tillering stage, the plasma membrane permeability in Hua-Hang-Si-Miao rice with Si treatment decreased by 56% (Cd + Si vs. Cd), 50% (Zn + Si vs. Zn) and 41% (Cd + Zn + Si vs. Cd + Zn).

Cd and excess Zn treatments caused a marked increase in MDA content in both cultivars during all three growth phases, but these increases were reduced by Si treatments (Figure 4b). In the seedling stage, the MDA concentration in the Feng-Hua-Zhan rice with Si treatment decreased by 39% (Cd + Si vs. Cd), 66% (Zn + Si vs. Zn) and 30% (Cd + Zn + Si vs. Cd + Zn).

### 3.4. Effect of Si on Total Chlorophyll Content and Chlorophyll Fluorescence Parameters

**Figure 5:**
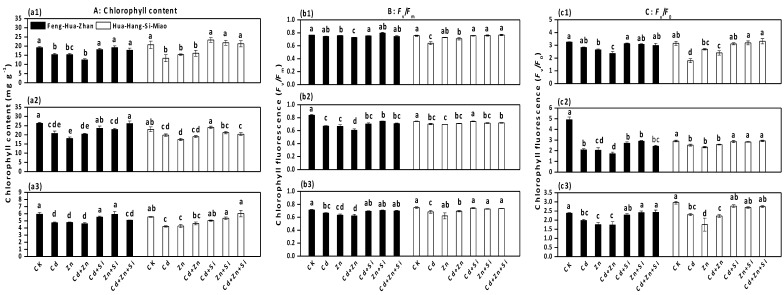
Effect of Si on the total chlorophyll content and the chlorophyll fluorescence parameter in two rice cultivars in different growth stages: (**a1**), (**b1**) and (**c1**) seedling stage; (**a2**), (**b2**) and (**c2**) tillering stage; (**a3**), (**b3**) and (**c3**) ripening stage. Error bars indicate standard deviations (*n* = 4). Bars with different letters are significantly different (*p* < 0.05) according to Duncan’s MRT.

A significant reduction was found in the total chlorophyll content under Cd and excess Zn treatments, but the decrease was mitigated by the application of Si (Figure 5a). The chlorophyll content under Si-treatments increased by 75% (Cd + Si vs. Cd), 42% (Zn + Si vs. Zn) and 32% (Cd + Zn + Si vs. Cd + Zn) in the seedling stage in the Feng-Hua-Zhan rice. Similar changes were observed in both rice cultivars in the tillering and ripening stages Cd and Zn caused a notable reduction in the *F*_v_/*F*_0_ ratio, but the reduction was mitigated by Si supplementation (Figure 5c). For example, in Hua-Hang-Si-Miao rice in the seedling stage (Figure 5c1), the values of *F*_v_/*F*_0_ increased by 73% in Cd + Si vs. Cd, 18% in Zn + Si vs. Zn and 38% in Cd + Zn + Si vs. Cd + Zn. Similar tends in response to *F*_v_/*F*_m_ were also observed, but no significant difference was found (Figure 5b).

### 3.5. Effect of Si on Antioxidant Enzymes Activities

**Figure 6:**
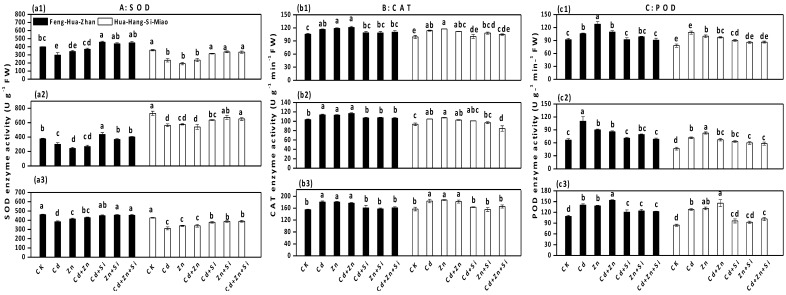
The effect of Si on antioxidant enzyme activity in two rice cultivars in different growth stages: (**a1**), (**b1**) and (**c1**) seedling stage; (**a2**), (**b2**) and (**c2**) tillering stage; (**a3**), (**b3**) and (**c3**) ripening stage. Error bars indicate standard deviations (*n* = 4). Bars with different letters are significantly different (*p* < 0.05) according to Duncan’s MRT.

SOD activities were significantly decreased under Cd and excess Zn stress at different growth stages compared to the control (Figure 6a). In the presence of Si, SOD activities significantly increased, especially for Feng-Hua-Zhan in the tillering stage which showed increases of 46% (Cd + Si vs. Cd), 52% (Zn + Si vs. Zn) and 49% (Cd + Zn + Si vs. Cd + Zn). In contrast, Cd and excess Zn significantly increased the CAT and POD activities in all cases (Figure 6b,c), but these increases were mitigated in plants grown in Si-treated soil. For example, for Feng-Hua-Zhan rice in the seedling stage, POD activities were decreased by 36% (Cd + Si vs. Cd), 12% (Zn + Si vs. Zn) and 20% (Cd + Zn + Si vs. Cd + Zn).

### 3.6. Effect of Si on the Cd and Zn Concentrations in Soil

**Figure 7:**
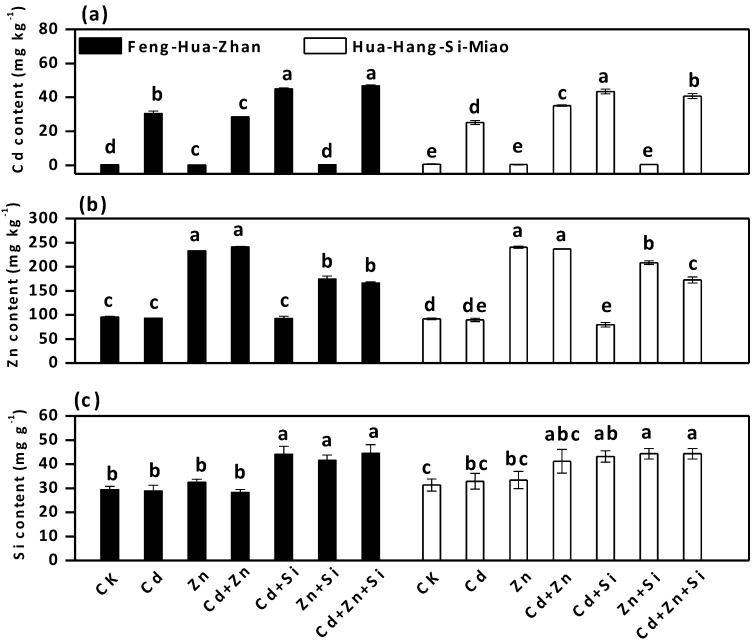
Effect of Si on available element concentrations in soil: (**a**) Cd content; (**b**) Zn content; (**c**): Si content. Error bars indicate standard deviations (*n* = 4). Bars with different letters are significantly different (*p* < 0.05) according to Duncan’s MRT.

Si treatments significantly reduced available Zn concentration in soil but increased available Cd concentration (Figure 7).

In Feng-Hua-Zhan, available Cd content increased by 48% (Cd + Si vs. Cd), but Zn concentration decreased by 25% (Zn + Si vs. Zn). As expected, available Si content in the Si-treated soils was significantly higher than that in the non-Si- treated soils.

## 4. Discussion

Si addition not only improves the growth of the rice plants, but it also reduces the toxic effects of Cd and Zn in both rice cultivars in the tillering and ripening stages, especially at a high Cd concentration of 50 mg·kg^−1^ (Table 1, Figure 1, Figure 2 and Figure 3). This finding was also supported by the observation of Si-mediated improvement of the photochemical parameters (Figure 5). These results are also in agreement with previous reports regarding the beneficial effect of Si on the plants treated with excess concentrations of elements such as As [31], Cr [32], Fe [33], Al [34] and Mn [35]. Moreover, Si application is attractive since abundant, inexpensive sources of Si-rich materials such as fly ash and steel slag [20], are readily available, and new silicon fertilizers such as Nano-Si fertilizer, are being developed [22]. In addition, the Si concentration in leaves was higher than those in any of the other plant organs after Si treatment (Figure 2), which is consistent with previous results obtained in rice [4], bamboo [36] and amaranth [37], indicating that Si is preferentially accumulated in the leaves. 

External and internal mechanisms have been cited by many researchers to explain the mitigating effect of Si on Cd toxicity in rice plants [9,38]. The available Cd concentration increased in soil (Figure 7) but significantly decreased in the shoots (stems, leaves and grains) after Si-treatment (Figure 2), which implied that Si-mediated mitigation may be due to internal mechanisms that limit Cd^2+^ uptake by the roots. This limitation effect can occur through passive adsorption in the apoplast or through active adsorption in the symplast [20,39,40]. Of these studies, Neumann and zur Neiden [39] demonstrated that Si-induced inhibition of symplastic transport was due to the sequestration of heavy metals in the cytoplasm. In addition, the inhibited growth of two rice cultivars was observed under a high Zn concentration of 200 mg·kg^−1^, but this negative effect was mitigated by the addition of Si (Table 1 and Figure 1). Similar effects were also reported by many researchers in maize [9,25,41], except for Bokor et al. [42], who reported that there was no positive effect of Si on excess Zn toxicity in the young maize hybrid under hydroponic conditions.

Exposure to these metals caused substantial damage to the plasma membrane of both rice cultivars, as reflected by a marked increase in electrolyte leakage (Figure 4). Similar results were obtained by Anwaar et al. [43] and Kaya et al. [25], who reported that excess Zn caused membrane damage in cotton and maize plants, respectively. The increases in MDA content were observed in all cases, suggesting that Cd and Zn caused oxidative damage to cell membranes in leaves. After Si addition, a marked reduction in the MDA content of both cultivars occurred during different growth phases, which indicated that heavy metal-induced oxidative stress was alleviated by Si. Similar effects were also reported in rice, peanuts and wheat subjected to Cd, Zn, Pb, As, and Mn stresses [11,33,44,45], showing the protective role of Si in preventing oxidative stress. Moreover, Liang et al. [46] reported that the Si application decreased the permeability of plasma membranes and membrane lipid peroxidation, which helped to maintain cell membrane integrity, stability and function. Thus, we propose that the protective effects of Si are attributable to the decrease in plasma membrane permeability and lipid peroxidation of leaf cells.

The *F*_v_/*F*_m_ values in both rice cultivars decreased under Cd and excess Zn stress (Figure 5), which might be attributed to the loss of efficiency of PSII related to photoinhibition [19]. Furthermore, the *F*_v_/*F*_0_ ratio reflects the activity of PSII that affects the efficiency of capturing excitation energy by an open PSII reaction center [27]. Cd and excess Zn inhibited the activities of PSII by decreasing the ratio, which was consistent with the results obtained in cucumber under Cd stress [27], in rice under high Zn stress [19], and in wheat under Cr(VI) stress [32]. In contrast, Si addition increased both values of *F*_v_/*F*_m_ and *F*_v_/*F*_0_ under Cd and excess Zn stress, which suggested that the metal-treated plants could regain a higher activity of reaction centers in the presence of Si [28]. This phenomenon has also been observed in rice under Cd stress [13] and in maize under excess Mn stress [47]. Additionally, significant Si-induced the enhancement of photosynthesis and was also reported in salt-stressed tomatoes [48] and drought-stressed sorghum [49]. Taken together, the positive effect of Si on photosynthesis might be due to the lower uptake of Cd and Zn, resulting in an increase in the chlorophyll content (Figure 5) and in the activity of PSII by reducing the damage to photosynthetic machinery (Figure 2).

Si treatment enhanced SOD activity in both rice cultivars during different growth phases (Figure 6), which suggested that Si may scavenge highly reactive oxygen species (ROS) that result from exposure to the metals. Similar results have been obtained by many researchers, most of whom reported that Si application caused a consistent increase in the SOD activity in roots of rice plants experiencing Zn toxicity [50], in the leaves of rice plants [22] and in the leaves of wheat plants experiencing Cd toxicity [10]. Under Cd and excess Zn stress, CAT and POD activities were increased in leaves, but Si addition reduced the activities of these anti-oxidant enzymes. This might be due to the removal of ROS in a non-enzymatic way by enhancing the activities of nonenzymatic anti-oxidants such as glutathione (GSH), non-protein thiols (NPT) and ascorbic acid (AsA) [51,52,53] or due to the enhancement of other anti-oxidant enzyme activities such as ascorbate peroxidase (APX) and SOD [50,54]. Similar phenomena have also been reported by other researchers [43,55,56], who observed that Si addition decreased POD or CAT activities in the leaves of cotton under Zn stress [43], in the leaves of cucumber under Mn stress [55], and in the leaves of peanuts under Al stress [56]. On the other hand, previous reports demonstrated that Si application enhanced the activities of POD and CAT in rice under Zn [50] and Cd stress [57]. These changes in the activity of anti-oxidant enzymes may be responsible for Si-related resistance to Cd and excess Zn toxicity in rice.

## 5. Conclusions

The present result clearly demonstrates the fact that Si application was able to reduce the phytotoxicity of Cd and excess Zn in rice plants, by decreasing membrane permeability and MDA contents, enhancing the photosynthetic activity and modulating anti-oxidant enzymes activities. Si treatment reduced Cd accumulation of rice plants through symplastic pathways in the root system. Moreover, Si-mediated mitigation exhibited a similar variation trend substantially in both rice cultivars during all three growth phases.

## Figures and Tables

**Table 1:** Effect of Si addition on shoot and root dry weights of rice under heavy metal stress.

Cultivars	Treatments	Tillering Stage		Ripening Stage
		Shoot (g pot^−1^)	Root (g pot^−1^)	Shoot (g pot^−1^)
Feng-Hua-Zhan	CK	47 ± 2.3^b^	6 ± 0.2^a^	83 ± 4.1^ab^
	Cd	42 ± 0.4^c^	5 ± 0.4^b^	57 ± 3.7^d^
	Zn	40 ± 0.8^c^	4 ± 0.4^b^	60 ± 0.7^d^
	Cd + Zn	48 ± 0.9^b^	6 ± 0.2^b^	56 ± 2.8^d^
	Cd + Si	54 ± 2.2^b^	7 ± 0.3^a^	73 ± 3.1^c^
	Zn + Si	51 ± 2.1^ab^	7 ± 0.6^a^	77 ± 2.7^bc^
	Cd + Zn + Si	54 ± 1.5^a^	7 ± 0.6^a^	91 ± 3.0^a^
Hua-Hang-Si-Miao	CK	47 ± 3.6^ab^	3 ± 0.2^cd^	145 ± 7.4^ab^
	Cd	41 ± 0.4^b^	3 ± 0.5^cde^	78 ± 5.3^d^
	Zn	38 ± 1.9^b^	2 ± 0.1^e^	82 ± 5.8^d^
	Cd + Zn	45 ± 2.6^ab^	2 ± 0.2^de^	94 ± 8.7^d^
	Cd + Si	45 ± 0.1^ab^	4 ± 0.1^c^	116 ± 4.4^c^
	Zn + Si	43 ± 1.8^ab^	5 ± 0.6^b^	132 ± 8.5^bc^
	Cd + Zn + Si	51 ± 4.7^a^	7 ± 0.5^a^	158 ± 5.2^a^

Note: *n* = 4, ±SE; Different letters in each column indicate significant differences.
